# EAHP 2020 workshop proceedings, pediatric myeloid neoplasms

**DOI:** 10.1007/s00428-022-03375-8

**Published:** 2022-07-11

**Authors:** R. J. Leguit, A. Orazi, N. Kucine, H. M. Kvasnicka, U. Gianelli, D. A. Arber, A. Porwit, M. Ponzoni

**Affiliations:** 1 Department of Pathology, University Medical Center Utrecht, H04-312, POB 85500, 3508 GA Utrecht, The Netherlands; 2 Department of Pathology, Texas Tech University Health Sciences Center El Paso, 4800 Alberta Avenue, El Paso, TX 79905 USA; 3 Department of Pediatrics, Division of Hematology/Oncology, Weill Cornell Medicine, 525 E. 68th St, Payson-695, New York, NY 10065 USA; 4 Institute of Pathology and Molecular Pathology, University of Witten/Herdecke, Witten, Germany; 5 Pathology Unit, Department of Pathophysiology and Transplantation, University of Milan and IRCCS Fondazione Ca’ Granda – Ospedale Maggiore Policlinico, Via Francesco Sforza 35, 20122 Milan, Italy; 6 Department of Pathology, University of Chicago, Chicago, IL USA; 7 Faculty of Medicine, Department of Clinical Sciences, Division of Oncology and Pathology, Lund University, Lund, Sweden; 8 Vita Salute San Raffaele University and Pathology and Lymphoid Malignancies Unit, IRCCS San Raffaele Scientific Institute, Milan, Italy

**Keywords:** Bone marrow biopsy, EAHP workshop, Pediatric, Myelodysplastic syndrome, Myeloproliferative neoplasm, Juvenile myelomonocytic leukemia, Acute leukemia

## Abstract

The first section of the bone marrow workshop of the European Association of Haematopathology (EAHP) 2020 Virtual Meeting was dedicated to pediatric myeloid neoplasms. The section covered the whole spectrum of myeloid neoplasms, including myelodysplastic syndromes (MDS), myeloproliferative neoplasms (MPN), myelodysplastic/myeloproliferative neoplasms (MDS/MPN), and acute myeloid leukemia (AML). The workshop cases are hereby presented, preceded by an introduction on these overall rare diseases in this age group. Very rare entities such as primary myelofibrosis, pediatric MDS with fibrosis, and MDS/MPN with JMML-like features and t(4;17)(q12;q21); *FIP1L1::RARA* fusion, are described in more detail.

## Introduction

Although less common in the pediatric age group, most myeloid neoplasms seen in adults can also occur in childhood where they may show differences from their adult counterparts. Some entities preferentially occur in childhood, such as several types of acute myeloid leukemia (AML) with specific recurrent genetic abnormalities, others exclusively in childhood such as juvenile myelomonocytic leukemia (JMML) and the provisional WHO entity refractory cytopenia of childhood (RCC). Furthermore, when dealing with children and young adults, one must be particularly aware of a possible underlying inherited or de novo germline mutation that might predispose to myeloid neoplasms.

In this paper, we present an overview of the neoplastic cases submitted to the EAHP 2020 bone marrow (BM) workshop on pediatric myeloid neoplasms. The cases were grouped into the following sections: myeloproliferative neoplasms (MPN), JMML and related diseases, myelodysplastic syndromes (MDS), and AML. Each section is preceded by an introduction, focusing on the specific characteristics in childhood, followed at the end by a summary highlighting the lessons learned from the workshop.

## Myeloproliferative neoplasms

### Chronic myeloid leukemia

Chronic myeloid leukemia (CML) is a *BCR::ABL1*-positive MPN, characterized by leukocytosis due to proliferation of mainly the granulocytic lineage. CML is rare in childhood, accounting for less than 10% of all CML cases and less than 3% of all pediatric leukemias. The median age at diagnosis in children is 11–12 years, with 60–77% occurring in males [1, 2].

The clinical presentation of CML in childhood is variable, with weakness and abdominal pain being the most common presenting symptoms, and some children being diagnosed incidentally [1, 2]. In comparison with adults, children tend to present with higher white blood cell (WBC) counts [3]. Splenomegaly is common, occurring in > 70%, whereby children tend to have a larger spleen size relative to body size than adults [2–4].

Pediatric CML frequently shows high to very high (> 1000 × 10^9^/L) platelet counts, without an increased risk of thrombosis [5]. Mild bleeding signs, however, are observed in 12% of children with thrombocytosis, which does not correlate to elevated platelet counts, but is associated with reduced high molecular weight multimers of von Willebrand factor, indicating an acquired von Willebrand syndrome. The latter resolves after initiation of CML treatment [5].

More than 90% of patients present in chronic phase. The proportion of pediatric patients diagnosed with advanced-stage disease (accelerated phase or blast phase) is, however, higher than that of adult patients [4]. Furthermore, in both children and adults, a molecular response with *BCR::ABL1* transcript levels of < 10% at 3 months after starting imatinib is associated with a better progression-free survival [3, 6], but a higher proportion of children fail to achieve this compared with adults (37% versus 24–28%) [3, 6].

CML at the pediatric age shows differences in genomic landscape compared with adult CML. In both children and adults, a hematopoietic stem cell has acquired the *BCR::ABL1* fusion gene and breakpoints occur in the same major breakpoint cluster regions (M-BCR) in the *BCR* gene on chromosome 22, but in chronic-phase CML the breakpoint distribution in *BCR* has been shown to be different in children [7]. Children show more frequent breakpoints in *Alu* repeat regions, and unlike adults with CML who have enrichment of fusion sites in the centromeric region, children show enrichment in fusion sites within telomeres overlapping with an *Alu* repeat region, similar to what is seen in adult Philadelphia-positive acute lymphoblastic leukemia with M-BCR rearrangement [4]. Additionally, transcriptomic analysis has demonstrated differentially expressed genes and pathways in childhood CML when compared to CML in adulthood [8].

Current prognostic scores used for adult patients (including the EUTOS, Hasford, and Sokal scores) are not validated in children. An attempt at validating these scoring systems showed a high discordance rate, confirming they cannot be applied to our youngest patients [4]. The recently proposed EUTOS Long-Term Survival score, which looks at age, spleen size below the costal margin, peripheral blast count, and platelet count, has been evaluated in 350 children and showed good differentiation of progression-free survival [9].

### Workshop cases

Four CML cases in chronic phase were submitted to the workshop (Table 1), of which three were girls (aged 17, 16, and 10 years) and one was a boy (3 years old). All showed the typical BM morphology of chronic-phase CML. Three presented with leukocytosis (WBC up to 521.1 × 10^9^/L), but one (case 730, the 16-year-old girl) showed a normal WBC of 5.6 × 10^9^/L and macrocytic anemia. Her karyotype {46,XX,t(9;22;1;6)(q34;q11.2;p12;q27), t(6;22)(p12;q12),der(9)t(5;9)(q13;q22)[10]/46,XX[2]} revealed a complex variant of the t(9;22) translocation, involving both chromosomes 9 and 22 as well as three other chromosomes (in addition to 9q34 and 22q11). FISH:BCR/ABL dual-color, dual-fusion probe was positive for t(9;22)(q34;q11.2) in 67.5% of cells with deletion on derivative chromosome 9. Standard and variant t(9;22) translocations in the current tyrosine kinase inhibitors era are thought to share a similar good prognosis.

**Table 1:** Chronic myeloid leukemia (CML) cases submitted to the EAHP 2020 workshop

**Case #**	**Submitter**	**Age** year	**Sex**	**Hb** g/dL	**WBC** × 10^9^/L	**Plt** × 10^9^/L	**Panel diagnosis**	**Remarks**
744	Dr. Ofori, New York, USA	17	F	9.0	242.9	501	**CML** **chronic phase**	*ABL1* TKI resistance mutations
743	Dr. Wooldridge, New York, USA	3	M	8.2	175	176	**CML** **chronic phase**	Response to TKI therapy
730	Dr. Wooldridge, New York, USA	16	F	4.7	5.6	214	**CML** **chronic phase**	Complex variant of the t(9;22) translocation
505	Dr. Liu, Pittsburgh, USA	10	F	7.4	521.1	646	**CML** **chronic phase**	Cryptic *BCR::ABL1* fusion. Normal karyotype, FISH*: BCR::ABL1* fusion; RT-PCR: *BCR::ABL1* major (p210)

*Hb* hemoglobin, *Plt* platelet count, *TKI* tyrosine kinase inhibitors, *WBC* white blood count

In CML, mutations in the kinase domain of the *BCR::ABL1* fusion gene may induce resistance to tyrosine kinase inhibitors, as was illustrated by case 744, containing multiple such mutations. On follow-up, this patient acquired an additional *EVI1* rearrangement, indicating clonal evolution.

Case 505 had a cytogenetically cryptic *BCR::ABL1* not detected by conventional karyotyping, but demonstrated by FISH and RT PCR, illustrating the need for additional investigation whenever the clinical and histological picture is suspicious for CML but conventional karyotyping is normal. Cryptic or masked *BCR::ABL1* fusions occur in 2–10% of CML patients.

### Myeloproliferative neoplasms, *BCR-ABL1* negative

The classical *BCR::ABL1*-negative MPNs, which include essential thrombocythemia (ET), polycythemia vera (PV), and primary myelofibrosis (PMF), are rare in children, with global incidences for children and young adults estimated to be around 0.6/100,000 patients/year (range 0.004 to 0.9) for ET, 0.18/100,000 patients/year for PV, and 0.53/100,000 patients/year (range 0.003 to 1.5) for PMF [11].

Thrombotic events are less common in pediatric than in adult MPN, whereas adults have an increased risk of both arterial and venous thrombosis, children and adolescents with ET and PV predominantly have venous events (84.2%), and hemorrhagic episodes are rare (< 5%) [11]. Thromboses of the splanchnic territories are most frequent (75% of the venous events), with a large predominance of Budd-Chiari syndrome (62.5% of all venous events) [11]. It has been suggested that the presence of a driver mutation may confer an increased risk of thrombosis in children with MPN [10]. Progression towards myelofibrosis and transformation into AML is very rare in pediatric ET and PV [11].

#### Essential thrombocythemia

Pediatric ET must be differentiated from reactive thrombocytosis and from the non-clonal hereditary thrombocytosis caused by several different germline mutations in the genes for thrombopoietin, thrombopoietin receptor (MPL), JAK2, and gelsolin [12, 13]. Most of these germline mutations show an autosomal dominant pattern of inheritance [12]. Although the majority of these mutations is not or only rarely associated with thrombotic or hemorrhagic events, the *JAK2* V617I, *JAK2* T875N, and *MPL* S505N mutation do show an increased risk for vascular events [14–16]. Furthermore, the *MPL* S505N mutation is associated with splenomegaly and progression to BM fibrosis, significantly affecting life expectancy [15].

In a systematic review on pediatric MPN, 396 children with ET are reported, showing a mean age at diagnosis of 9.3 years [11]. In this study, about half of the patients were asymptomatic at diagnosis, with headache being the most frequent symptom, present in 27.5% of patients, and splenomegaly being the most frequent abnormal sign, occurring in 55% [11]. In this systematic review, A *JAK2* V617F mutation was found in 31%, a *CALR* mutation in 10%, and a *MPL* mutation in 2% of patients [11], showing a much higher rate of triple negative disease (57%) than is reported in adults with ET (12%) [17].

#### Polycythemia vera

*JAK2*-negative PV cases must be differentiated from reactive polycythemia and from hereditary erythrocytosis. Hereditary erythrocytosis can be secondary due to, among others, mutations in genes in the oxygen sensing pathway, the erythropoietin (EPO) gene, or genes encoding high-affinity hemoglobins, and are associated with elevated or normal EPO levels [18]. In primary erythrocytosis, caused by mutations affecting erythroid progenitor cells, patients may, however, have low EPO levels, elevated hematocrit, and erythroid progenitors being hypersensitive to EPO, thereby mimicking PV [19]. Primary hereditary causes are very rare, the most common being mutations in the gene encoding the erythropoietin receptor (*EPOR*) [18, 19].

The mean age at diagnosis for pediatric PV is 11–12 years [11, 20]. Up to half of pediatric PV patients were reported to be asymptomatic at diagnosis, with headache being the most frequent symptom, reported in 30.5% to up to 100% of PV patients in some studies [11, 20, 21]. Thrombotic events occur in 15–25% of children, mainly in the form of Budd–Chiari syndrome [11, 20]. The presence of marked to severe leukocytosis (> 15 × 10^9^/L) seems to be associated with both thrombotic and hemorrhagic complications [20]. In the largest study on PV, encompassing 75 children, splenomegaly was the most frequent abnormal sign, occurring in 15% of young PV patients [11]. A *JAK2* V617F or *JAK2* exon 12 mutation was demonstrated in this large study in only 37% and 2.5% of pediatric PV patients, respectively. The authors of this systematic review appropriately questioned whether these reported PV patients were properly diagnosed with PV and suggested that confirmation of this finding with a more recently diagnosed cohort could be helpful [11].

A recurring issue in MPN diagnosis is the separation of ET from PV particularly “masked” PV. As pediatric marrows already physiologically display high cellularity, determination of “hypercellularity” is difficult and the parameter of cellularity therefore usually is not contributory in separating ET from PV. Furthermore, lack of iron deposits is common in pediatric marrows and therefore this parameter does not discriminate PV from ET. To rule out PV, it is important referring to age-adjusted values (see, e.g., Nathan and Oski’s Hematology of Infancy and Childhood) for hemoglobin (Hb) and hematocrit (Hct) [22], particularly in patients less than 10 years old. A very low EPO level favors PV over ET. However, a normal EPO value does not rule out PV [20].

#### Primary myelofibrosis

Pediatric PMF is very rare. The two largest studies on pediatric PMF are one from the Texas Children’s Hospital, USA, describing 19 children, and one from China, describing 14 children with PMF [23, 24]. Median age at onset is very different between the two studies: 14 months (range 0–17 years) and 13.5 years (range 2–18 years) [23, 24]. Boys were affected slightly more than girls [23, 24]. Almost all patients had anemia, more than 85% showed thrombocytopenia, and 37–64% displayed neutropenia [23, 24]. The frequency of splenomegaly and hepatomegaly ranged between the studies from 21 to 63% for splenomegaly and 0 to 53% for hepatomegaly, with the lowest rates in the Chinese study [23, 24]. In the study from Texas, the amount of fibrosis varied, being MF1 (5 patients), MF2 (12 patients), or MF3 (2 patients). Megakaryocytic changes were described in this study as nuclear hypolobulation, separation of nuclear lobes, and the presence of micromegakaryocytes [23], rather than the presence of hyperchromatic, bulbous or “cloud-like”-shaped nuclei typically described in adult cases of PMF. *JAK2* V617F and *MPL* W151L were tested in 17 and 6 children, respectively, but no mutations were found. Five cases had spontaneous resolution, but as no mutations were found, a reactive process in these cases cannot be ruled out. Although none showed acute leukemic transformation, 8 children died, half of them after hematopoietic stem cell transplantation (SCT) [23]. In the study from China, patients had either MF2 (3 patients) or MF3 (11 patients), and a *CALR* type 2 mutation was detected in 50% of patients [24]. Mutations of *JAK2* V617F or *MPL* W515K/L were absent. In this study, none of the patients had spontaneous remission and six patients (43%) transformed to AML [24].

### Workshop cases

#### Essential thrombocythemia

**Fig. 1:**
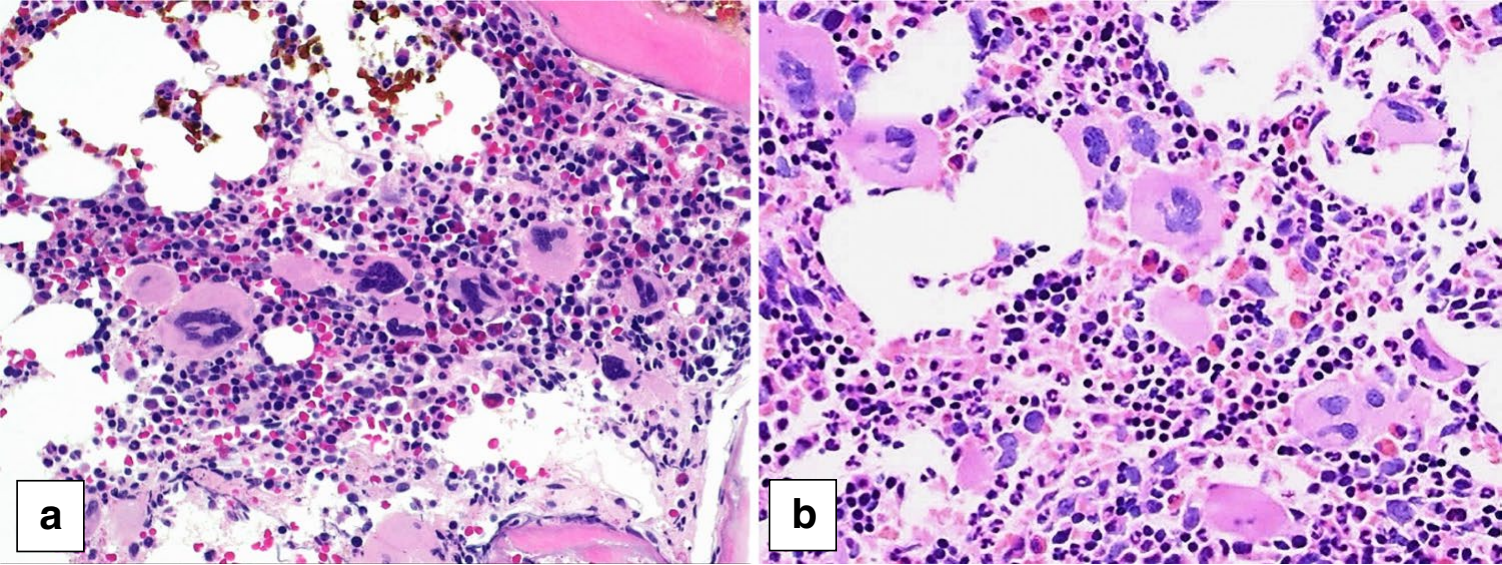
Pediatric bone marrow biopsies with essential thrombocythemia showing large hyperlobulated megakaryocytes (**a** case 153, **b** case 564)

Of the 11 pediatric *BCR::ABL1*-negative MPN cases submitted to the workshop, 4 were classified by the panel as ET (Table 2). BM morphology was in all cases similar to that of adult ET with enlarged, hyperlobulated megakaryocytes (Fig. 1). Case 722 describes a 17-year-old male presenting with micturition problems and gastrointestinal bleeding. He was found to have thrombocytosis of 800–900 × 10^9^/L and was diagnosed with a triple negative ET. The second ET case (case 408) describes an 11-year-old girl with pruritus, thrombocytosis of 1500 × 10^9^/L and a *CALR* exon 9 mutation. The third ET case (case 153) describes a 17-year-old girl with chest pain, neck pain, and tingling of the arms for 2 weeks. She was found to have thrombocytosis of 1022 × 10^9^/L and a *MPL* W515K mutation. The fourth ET case (case 564) was that of a 15-year-old girl who presented with headache. She had a 10-year history of thrombocytosis, most recently of 1100 × 10^9^/L. A *JAK2* V617F mutation was detected.

**Table 2:** *BCR-ABL*-negative myeloproliferative neoplasms (MPNs) submitted to the EAHP 2020 workshop

**Case #**	**Submitter**	**Age** year	**Sex**	**Hb** g/dL	**WBC** × 10^9^/L	**Plt** × 10^9^/L	**Splenomegaly**	**Thrombotic events**	**Driver mutation**	**Panel diagnosis**
722	Dr. Own, Stockholm, Sweden	17	M	15.7	6.0	*800–900*	Yes	–	–	**ET**
408	Dr. Uner, Ankara, Turkey	11	F	14.6	8.3	*1500*	Yes	–	*CALR* exon 9	**ET**
153	Dr. Grier, Cincinatti, USA	17	F	14.3	11.0	*1022*	Yes	–	*MPL* W515K	**ET**
564	Dr. Kwiecinska, Stockholm, Sweden	15	F	15.0	*13.0*	*1100*	Yes	–	*JAK2* V617F	**ET**
602	Dr. Gheorghe, Minnesota, USA	7	F	*17.2*	*16.4*	*826*	Yes	–	*JAK2* V617F (5%)	**PV**
496	Dr. Mays, New York, USA	19	M	*14.4*	NL	*1000*	Yes	Chronic portal vein thrombosis, pulmonary emboli	*JAK2* V617F	**PV**
700	Dr. Grier. Cincinnati, USA	9	F	*15.1*	*42.8*	157	Yes	–	*JAK2* V617F (61%)	**PV in accelerated phase**
678	Dr. Orazi, Texas, USA	5	M	**10.5**	*11–23*	*939*	No	–	*CALR* type 1	**Pre-PMF**
627	Dr. Burroni, Paris, France	13	F	11.5	12.0	*1300*	Yes	Jugular and subclavian vein thrombosis	–	**Pre-PMF**
182	Dr. Margolskee, Philadelphia, USA	17	F	12.0	12.0	*816*	Yes	Venous sinus thrombosis	*MPL* inframe insert (15%)	**PMF MF2**
679	Dr. Schafernak, Phoenix, USA	15	M	NL	*34.3*	*1834*	Yes	–	–	**MPN-U,** **favor pre-PMF**

Blood values in italics are increased and that in bold decreased according to the age adjusted values of the local laboratory

*ET* essential thrombocytopenia; *Hb* hemoglobin; *NL* normal; *Plt* platelet count; *PMF* primary myelofibrosis; *pre-PMF* prefibrotic PMF; *MPN-U* myeloproliferative neoplasm, unclassifiable; *PV* polycythemia vera; *WBC* white blood count

None of the four ET patients experienced thrombotic events. Three out of four cases showed mild splenomegaly (range 13.1–15.2 cm), which is consistent with previous reports that children with ET often show splenomegaly [11]. Only case 153 showed fibrosis (MF1).

Based on our workshop case submissions, it seems that the percentage of triple negative ET cases may be lower than what was reported in some studies [11], but not in others [25]. The observed variability may reflect whether reactive or constitutional etiologies were carefully excluded.

#### Polycythemia vera

Three pediatric PV cases were submitted to the workshop. In all three cases, a *JAK2* V617F mutation was detected (Table 2).

The first case (case 602) describes a 7-year-old girl with a 3-year history of chronic headaches, epigastric abdominal pain, and constipation. She also had a 3-year history of persistent leukocytosis (WBC now 16.4 × 10^9^/L) with neutrophilia (neutrophils 72%) and thrombocytosis (826 × 10^9^/L), with recently additional erythrocytosis (Hb 17.2 g/dL; Hct 51.1%). EPO levels were low. She had splenomegaly and her *JAK2* V617F mutation allelic burden was 5%. She had no thrombotic events. The BM biopsy showed a hypercellular marrow (cellularity 90%) with panmyelosis. Megakaryocytes were increased and showed pleomorphic features with occasional loose clusters.

**Fig. 2:**
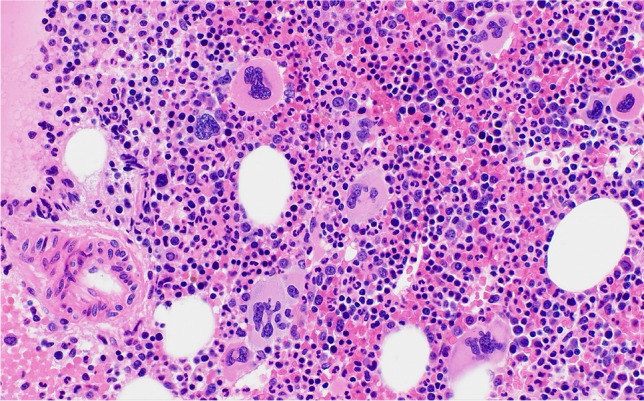
Bone marrow biopsy of a 19-year-old young man with panmyelosis and increased, pleomorphic megakaryocytes, consistent with polycythemia vera (case 496)

The second PV case (case 496) describes a 19-year-old boy who was diagnosed at the age of 2 (year 1995) with carnitine deficiency. Concurrent blood abnormalities led to a clinical diagnosis of PV. A BM biopsy performed in 1997 showed increased pleomorphic megakaryocytes, consistent with the diagnosis PV (Fig. 2). Complete blood counts 1 year later showed polycythemia according to the local age adjusted reference values (Hb 14.4 g/dL, elevated Hct) as well as thrombocytosis (1000 × 10^9^/L), undetectable EPO levels, and elevated LDH (613 U/L). In 2004, at the age of 10, his blood values deteriorated and he developed splenomegaly (16.1 cm). Pegylated interferon treatment was started at this point, on which he showed hematologic response and decrease in spleen size. In 2012, a *JAK2* V617V mutation had been demonstrated. Unfortunately, he developed chronic portal vein thrombosis (in 2014) and pulmonary embolisms (in 2015). Whether or not his carnitine deficiency played a role in his thrombotic events remains undetermined.

**Fig. 3:**
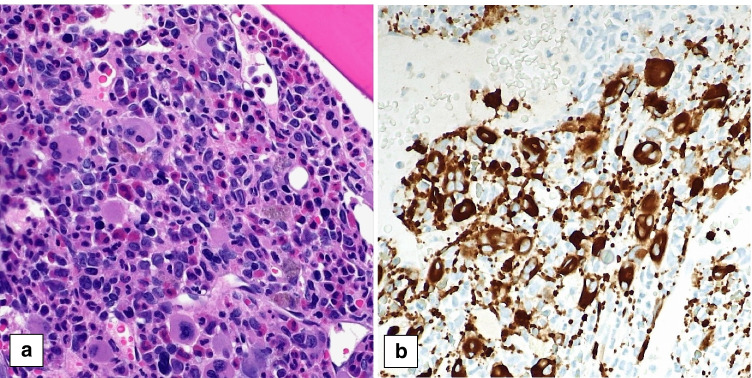
Bone marrow biopsy of a 9-year-old girl with polycythemia vera in accelerated phase (case 700). The HE stain (**a**) shows increased left shifted granulopoiesis and erythropoiesis, the latter showing megaloblastoid changes, associated with increased megakaryopoiesis characterized by the presence of a mixture of large and small dysplastic forms as highlighted in the CD61 stain (**b**). Her peripheral blood smear showed 4% blasts and her BM aspirate contained 14% blast by cytology and 13% myeloblasts by flow cytometry

The third PV case (case 700) describes a 9-year-old girl who presented with a large bruise on her left calf in association with weight loss and fatigue. No thrombotic events were reported. She was found to have splenomegaly (6 cm below the costal margin). Laboratory investigation revealed erythrocytosis (Hb 15.1 g/dl, Hct 57.4%, RBC 7.8 × 10^12^/L, MCV 73.9 fL) and leukocytosis (42.8 × 10^9^/L), but normal platelet count (157 × 10^9^/L). Peripheral blood (PB) smear showed 4% blasts. The BM aspirate had 14% blast by cytology; 13% myeloblasts were detected by flow cytometry. Ancillary studies demonstrated a *JAK2* V617F mutation (variant allele frequency (VAF): 61%), as well as a *JAK2* R867Q mutation (VAF: 5%), *RUNX1* R204Q mutation (VAF: 45%), and *IKZF1* loss. The BM biopsy showed hypercellular marrow (> 90% cellularity) with increased trilineage hematopoiesis and focally increased blasts (Fig. 3a). Megakaryocytes were pleomorphic with a predominance of smaller forms noticed (Fig. 3b). MF1 fibrosis was present. Based on the increased Hb and Hct (and of RBC), the presence of a low MCV, the high frequency of *JAK2* mutation, and the increase of blasts, a diagnosis of PV presenting in accelerated phase was made. She underwent cytoreduction with ruxolitinib followed by a matched unrelated donor SCT.

As all the three submitted PV cases were *JAK2* positive, it seems that, similar to ET, the percentage of triple negative PV pediatric cases might be lower than previously published by others [11]. This is supported by a yet unpublished study from Weill Cornell Medical Center in which all children diagnosed with PV demonstrated a *JAK2* mutation, as was presented at the virtual EAHP 2020 meeting by Dr. Kucine.

#### Primary myelofibrosis

Three cases of PMF were submitted to the workshop, two of which presenting in the prefibrotic stage (pre-PMF) (Table 2).

**Fig. 4:**
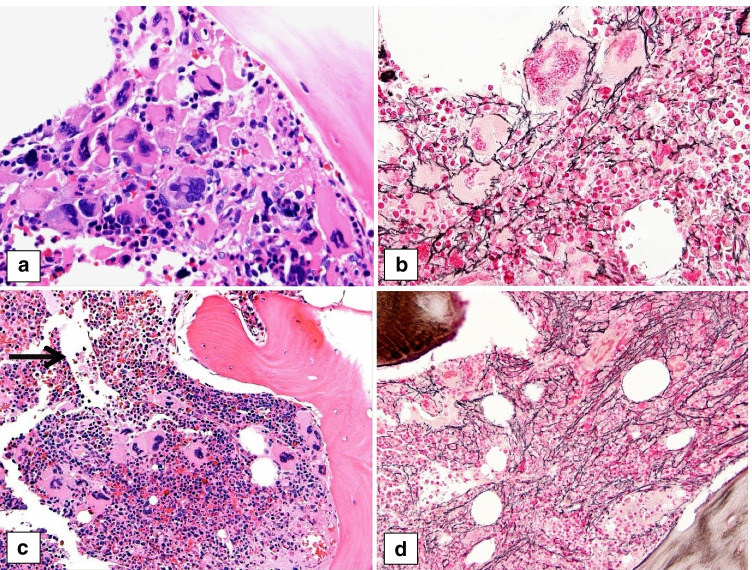
Bone marrow biopsies of a 5-year-old boy with primary myelofibrosis (PMF) with a *CALR* type 1 mutation (case 678), presenting in the prefibrotic stage and showing progression to the fibrotic stage. The first biopsy (**a**) shows tight clusters of markedly atypical megakaryocytes, including hypolobulated cloud-like and hyperchromatic forms, consistent with PMF. The reticulin stain (**b**) shows only mild fibrosis, MF1, consistent with the prefibrotic stage. A follow-up biopsy (**c**) shows hypercellular marrow with increased granulopoiesis, decreased erythropoiesis, tight clusters of atypical, hyperchromatic megakaryocytes and dilated sinusses (arrow). The reticulin stain (**d**) shows moderate fibrosis, MF2, consistent with progression to fibrotic stage PMF

The first case (case 678) is that of a 5-year-old boy with a history of frequent upper respiratory infections, significant dental caries, and self-limited bloody stools. He was found to have thrombocytosis (range 889–1147 × 10^9^/L), mild normocytic anemia (10.5 g/dL), and neutrophilic leukocytosis (WBC 11–23 × 10^9^/L, 95% neutrophils). LDH was high. There was no splenomegaly. A *CALR* type 1 mutation was demonstrated. The first BM biopsy showed normocellular to focally hypercellular (> 90%) marrow with increased granulopoiesis and megakaryopoiesis, and decreased maturing erythropoiesis. The megakaryocytes showed cytological atypia with cloud-like/bulbous and often hyperchromatic nuclei with large tight clusters (Fig. 4a), similar to PMF seen in adults. This initial biopsy showed only mild fibrosis (MF1) consistent with pre-PMF (Fig. 4b), but a follow-up biopsy demonstrated progression to overt myelofibrosis (PMF-MF2) (Fig. 4c, d).

**Fig. 5:**
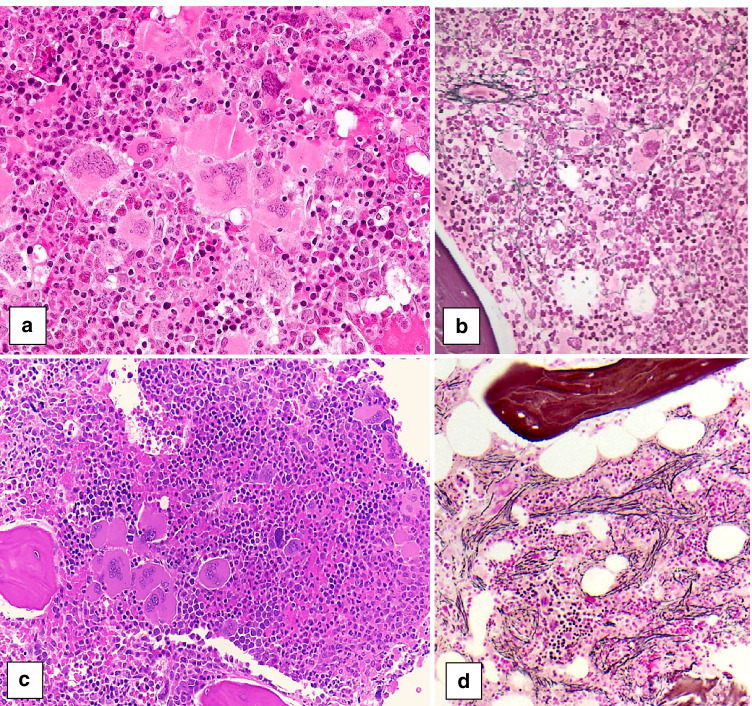
Two cases of pediatric primary myelofibrosis (PMF). **a**, **b** Bone marrow biopsy of a 13-year-old girl with a triple negative prefibrotic PMF (case 217) showing hypercellular marrow with tight clusters of atypical megakaryocytes (**a** HE) and no fibrosis (**b** reticulin). **c**, **d** Bone marrow biopsy a 17-year-old girl with a *MPL* positive PMF (case 182) showing hypercellular marrow with tight clusters of atypical megakaryocytes (**c** HE) and moderate fibrosis, MF2 (**d** reticulin), consistent with PMF, fibrotic stage

The second PMF case (case 627) describes a 13-year-old girl presenting with headache and ear pain. Laboratory investigation revealed marked thrombocytosis (2000 × 10^9^/L) and elevated LDH (340 U/L), but normal Hb and WBC. Splenomegaly (17 cm) was detected. No thrombosis was apparent. On treatment with hydroxyurea, platelet counts dropped. When hydroxyurea was stopped, platelet counts increased and neutrophilic leukocytosis developed (12 × 10^9^/L with 85% neutrophils). The BM biopsy showed a hypercellular marrow characterized by a marked megakaryocytic proliferation with large forms displaying hypolobulated, cloud-like shaped hyperchromatic nuclei as well as some forms with hyperlobulated nuclei (Fig. 5a). Loose as well as tight clusters of megakaryocytes were present. There was no fibrosis (Fig. 5b) and no increase in blasts. The features in the marrow were consistent with pre-PMF. Molecular studies showed no driver mutation or any other mutation in the 45 genes NGS panel. On follow-up, the patient developed jugular and subclavian thrombosis, on which she was started on heparin, vitamin K antagonists, and pegylated interferon. Later, she underwent hematopoietic SCT from a tissue-matched donor with good outcome.

The third PMF case (case 182) describes a 17-year-old girl presenting with severe headaches. Five years earlier, she had similar symptoms and cerebral venous sinus thrombosis. Anti-cardiolipin IgG was elevated at the time and she was diagnosed with anti-phospholipid syndrome, which was treated with warfarin and aspirin. Repeated imaging revealed again venous sinus thrombosis, but also splenomegaly. Laboratory investigation showed leukocytosis (12.0 × 10^9^/L), thrombocytosis (816 × 10^9^/L), and elevated LDH (1216 U/L). Hb and EPO levels were normal. There was no leucoerythroblastic picture in the blood. The BM biopsy showed hypercellular marrow with increased granulopoiesis and megakaryopoiesis, and adequate erythropoiesis. The megakaryocytes were often large with bulbous nuclei and tight clustering, consistent with PMF (Fig. 5c). Patchy but overall moderate fibrosis (MF2) was present (Fig. 5d), consistent with fibrotic stage PMF. A *MPL* mutation with in-frame insertion was demonstrated (VAF: 15%).

All three submitted cases showed the typical histopathological features of PMF as seen in adults, suggesting that morphological criteria for PMF as described in the revised 2016 WHO classification might also be applicable for children.

#### MPN, unclassifiable

One pediatric MPN case (case 679) could not be further classified by the panel (Table 2). It describes a 15-year-old boy with a history of migraine, presenting with lower back and penile pain, and intermittent chest pain for the past several days. He was found to have thrombocytosis of 1834 × 10^9^/L and leukocytosis of 34.3 × 10^9^/L, and mild splenomegaly (18.8 cm). There was no anemia and the PB smear showed adequate normocytic/normochromic red blood cells. No MPN driver mutation or other mutation was demonstrable. The BM was slightly hypercellular with a megakaryocytic proliferation with enlarged, slightly clustered megakaryocytes, consistent with an MPN. There was no fibrosis. Based on BM morphology and marked leukocytosis, the panel felt that this case of triple negative MPN most likely represented pre-PMF (rather than ET).

## Juvenile myelomonocytic leukemia and related diseases

### Juvenile myelomonocytic leukemia

JMML is characterized by a proliferation of the granulocytic and monocytic lineages and develops at the age of 0 to 14 years, with a median age of 1.3–2 years, 95% occurring under the age of 5 [26–28]. Boys are affected about twice as frequent as girls [26, 27]. Most patients present with constitutional symptoms or signs of infections due to cytopenias and leukemic infiltration into organs [27]. Besides splenomegaly, hepatomegaly is almost always present and lymphadenopathy is common [27]. A skin rash is present in about one-third of cases [27, 28]. Hemoglobin F (HbF) is often elevated for age [28]. The PB values and PB cytology are the most important for diagnosis. BM findings alone are not diagnostic.

The majority (~ 85%) of JMML cases has a driver mutation in one of the genes of the canonical RAS pathway, most frequently in *PTPN11*, *NRAS*, *KRAS*, *NF1*, or *CBL* [26, 29, 30]. *PTPN11*, *NRAS*, *KRAS*, and *CBL* mutations can be either germline or somatic, whereas *NF1* mutations are always germline [31–34]. The germline mutations generally occur as part of a broader syndrome (see RASopathies below), which carry an increased risk for developing JMML [31, 35].

A second, often subclonal, mutation occurs in less than half of JMML cases; this mutation can occur in another RAS pathway gene or in *SETBP1*, *JAK3*, *SH3BP1*, *SH2B3*, or components of the polycomb repressive complex 2, including *EZH2* and *ASXL1* [29, 30, 36]. Monosomy 7/del(7q) is the most common cytogenetic abnormality, present in 25–33% of cases [26–28].

The clinical course of JMML is generally aggressive, but mild forms or transient JMML-like myeloproliferations can occur, e.g., in the setting of a RASopathy or in the form of a RAS-associated autoimmune leukoproliferative disorder (RALD) as is discussed below.

### RASopathies

Part of the JMML cases occur in the setting of a RASopathy. The RASopathies form a group of autosomal dominant neurodevelopmental dysmorphic syndromes caused by a germline mutation in genes that encode components of the RAS/MAPK pathway, which are detected in approximately 70–75% of patients [31]. They are associated with autoimmunity; 14% has a clinical diagnosis of an autoimmune disease and in 52% of patients, autoimmune antibodies can be demonstrated [37, 38]. The two most common RASopathies, Noonan syndrome and neurofibromatosis type 1, as well as CBL syndrome, one of the more rare Rasopathies also known as “Noonan-syndrome-like disorder with or without juvenile myelomonocytic leukemia,” predispose to JMML [32].

Noonan syndrome is the most common RASopathy and characterized by distinctive facial dysmorphisms, congenital heart defects and cardiomyopathy, reduced postnatal growth, and variable cognitive deficits [39]. A germline *PTPN11* mutation is present in 40–50% of patients [40]. The remainder of the germline mutations occur in more than 10 other genes of the RAS/MAKP signaling pathway. A bona fide JMML is seen in 3% of children with *PTPN11* mutated Noonan syndrome, often with an excess of blasts and very severe neonatal manifestations, half of patients dying in the first month of life [41]. JMML has occasionally been described in *KRAS* mutated Noonan syndrome [42]. A transient JMML-like myeloproliferative disorder is seen in 2.5–10% in infants with Noonan syndrome, almost invariably harboring a germline *PTPN11* mutation [41, 43, 44].

Approximately 10–14% of JMML cases occur in children with neurofibromatosis type 1 [27], containing a germline mutation in the *NF1* gene encoding for neurofibromin [45]. Children with neurofibromatosis type 1 and JMML tend to be older and show a more aggressive form of the disease [27].

The percentage of germline *CBL* mutations detected in JMML patients varies up to 17% in the largest studies [26, 46–49]. Most of these patients show constitutional anomalies consistent with CBL syndrome, which include Noonan-like features (developmental delay, congenital heart defects, and craniofacial anomalies), neurologic deficits, and pigmented skin lesions [34, 48, 50, 51]. CBL syndrome carries an increased risk for JMML. Whereas the few recently described cases of JMML with a somatic *CBL* mutations were refractory to chemotherapy [34], the clinical course of JMML with a germline *CBL* mutation is heterogeneous with some cases showing spontaneous regression and others behaving aggressively [34, 50, 51].

### Workshop cases

Several challenging cases of JMML and related disorders were submitted to the workshop (Table 3).

**Table 3:** Juvenile myelomonocytic leukemia (JMML) and related disorders submitted to the EAHP 2020 workshop

**Case no**	**Submitter**	**Age**	**Sex**	**Hb** g/dL	**WBC** × 10^9^/L	**Mono** × 10^9^/L and/or %	**Plt** × 10^9^/L	**Genetic aberrations**	**Panel diagnosis**	**Remarks**
**521**	Dr. Gong, Chicago, USA	3 years	F	*8.8*	*32.3*	*5.8*	*69*	*RRAS2* (pQ72L)-germline *JAK3* (p.R657Q)	**JMML**	
**279**	Dr. Wang, Los Angeles, USA	5 years	M	*6.7*	*5.1*	*1.07*	*13*	*PTPN11* (46%) delY	**JMML**	Low WBC but absolute monocytosis
**492**	Dr. Kaumeyer, Chicago, USA	5 months	M	*7.1*	*41*	*12.6/20%*	*105*	*NF1* c.2033dup pI679Dsa*21 (75%) del7	**AML-MRC**	Possibly evolving from an underlying JMML. PB: 19% blasts
**344**	Dr. Zimmermann, Cincinnati, USA	4 years	M	*9.5*	*1.4*	*n/p*	*24*	*PTPN11* G503V	**AML-MRC ex JMML vs MPAL**	Probably clonally unrelated. Status post SCT for JMML. BM ~ 30% blasts
**471**	Dr. Petrova-Drus, New York, USA	9 months	M	*8.8*	*45.9*	*14%*	*149*	*FIP1L1::RARA* fusion t(4;17)(q12;q21)	**MDS/MPN with JMML-like features and t(4;17)(q12;q21); *FIP1L1::RARA* fusion**	Increased HbF, splenomegaly
**472**	Dr. Hoang, Los Angeles, USA	16 mo	F	*5.2*	*156.4*	*n/p*	*109*	*FIP1L1::RARA* fusion t(4;17)(q12;q21),t(2;3), add(7)(p21),del(9)(p21)	**MDS/MPN with JMML-like features and t(4;17)(q12;q21); *FIP1L1::RARA* fusion**	
**473**	Dr. Fang, Houston, USA	5 years	F	*nl*	*21.4*	*12%*	*nl*	*KRAS* G13C (37%)	**RAS-associated leukoproliferative disease (RALD)**	Germline RASopathy (non-malignant)

*AML-MRC* acute myeloid leukemia with myelodysplasia-related changes; *Hb* hemoglobin; *MDS/MPN-U* myelodysplastic/myeloproliferative neoplasm, unclassifiable; *MPAL* mixed phenotype acute leukemia; *n/p* not provided; *nl* normal; *PB* peripheral blood; *Plt* platelet count; *SCT* stem cell transplantation; *WBC* white blood count

#### JMML and RASopathies

Case 521 describes a 3-year-old girl with fever, showing typical JMML-associated features including leukocytosis, monocytosis, anemia, thrombocytopenia, splenomegaly, and elevated HbF (56.5%). The presence of a germline *RRAS2* (pQ72L) mutation, a gene in the RAS/MAKP pathway, further confirmed the diagnosis of JMML. She had no clinical characteristics of a RASopathy. *RRAS2* is one of the less frequently mutated genes in JMML and up to now has not been associated with Noonan syndrome, the most common RASopathy [52]. In addition, this patient had a somatic *JAK3* (p.R657Q) mutation. *JAK3* mutations in JMML are always secondary and found in distinct subclones, present in 2.8–12% of cases [29, 30]. *JAK3* mutations are associated with mutated *PTPN11* or *RAS* [29, 30], and associated with poor prognosis and progression of disease [30].

Case 279 describes a 5-year-old boy with a borderline-low leukocyte counts (WBC 5.1 × 10^9^/L), monocytosis (1.07 × 10^9^/L), severe anemia, thrombocytopenia, rare (~ 1%) circulating blasts, splenomegaly, and a somatic *PTPN11* mutation. Although WBCs were low, there was absolute monocytosis, so that the diagnostic criteria for JMML were still met.

Case 492 describes a 5-month-old boy with AML with myelodysplasia-related changes (AML-MRC) who presented with decreased feeding and upper respiratory infections. He had leukocytosis, monocytosis, anemia, thrombocytopenia, and marginally elevated HbF (21.7%) as well as 19% circulating blasts. In addition, he was shown to have splenomegaly. BM showed trilinear dysplasia and 25–30% blasts. Monosomy 7 and an *NF1* mutation (VAF: 75%) were documented. The peripheral blood findings (leukocytosis and monocytosis), the elevated HbF, and the presence of splenomegaly in association with *NF1* mutation and monosomy 7 suggested the possibility of an underlying JMML that evolved to AML-MRC.

Case 344 describes a 4-year-old boy who underwent allogeneic SCT for JMML, who presented with acute leukemia 5 months thereafter. Flow cytometry of the BM revealed a mixed population of myeloid blasts (~ 30%) and progenitor B cells (~ 30%), raising the possibility of mixed phenotype acute leukemia (MPAL). FISH study detected a deletion 7q in 96% of cells. In the differential diagnosis, AML-MRC ex JMML is considered because of the 7q deletion, but the possibility of a therapy-related myeloid neoplasm is also possible despite the short time interval. Without further molecular data to assess whether the AML and JMML were clonally related, this differential diagnosis could not be resolved.

#### RAS-associated autoimmune leukoproliferative disorder

Case 473 describes a now 34-year-old woman with a history of pancytopenia since age 2. At the age of 5, she underwent splenectomy because of massive splenomegaly. She also had a history of rheumatoid arthritis and possible autoimmune lymphoproliferative syndrome (ALPS). At age 34, she presented to the hospital with diffuse lymphadenopathy as well as diffusely hypermetabolic BM uptake on PET-CT. CBC showed leukocytosis (21.4 × 10^9^/l) with monocytosis (12%). Hb and platelet counts were within normal range. Due to these findings, BM and lymph node biopsies were performed. The BM biopsy and smears showed a hypercellular (90%) marrow with myeloid predominant trilineage hematopoiesis and increased monocytes. There was mild dysplasia within granulocytes and megakaryocytes. Plasma cells were slightly increased with occasional large forms. The lymph node biopsy showed a picture consistent with Rosai-Dorfman disease. In lymph node and BM, the same *KRAS* G13C mutation was detected.

The indolent course of the disease in the above case is most compatible with the entity called RAS associated autoimmune leukoproliferative disorder (RALD). Whereas JMML with *KRAS* or *NRAS* mutations generally run an aggressive course, RALD shows an indolent clinical behavior and long-term survival in absence of therapy [53]. As in the case described, RALD has many clinical and laboratory features overlapping with JMML, typically presenting with persistent absolute or relative monocytosis, massive splenomegaly, and lymphadenopathy, and is associated with autoimmunity [54, 55].

HbF can be increased for age [55]. Patients often present in childhood, frequently within the first year of life [55]. Almost all RALD patients meet the diagnostic criteria for JMML, but the accumulation of additional genetic abnormalities in JMML cells seems to contribute to the malignant phenotype in the latter [55]. RALD also shares many features with ALPS, including lymphadenopathy, massive splenomegaly, increased circulating B cells, hypergammaglobulinemia, and autoimmune cytopenia, but germline or somatic mutations in *FAS, FASL*, or *CASP10* characteristic for ALPS are absent in RALD. In addition, the CD4-/CD8-double-negative T cell receptor αβ T cells typical for ALPS, are present at normal levels or only marginally elevated in the peripheral blood and absent in lymph nodes. As there are no molecular data on the possible ALPS of case 473 described above, it might well be that what was previously taken for ALPS might retrospectively already have been RALD given the many clinical similarities of these two entities. RALD with an associated Rosai-Dorfman disease has been previously described in a 15-year-old boy with the same *KRAS* G13C mutation as in the submitted case [56].

#### MDS/MPN with JMML-like features and t(4;17)(q12;q21); *FIP1L1::RARA* fusion

Two cases (case 471 and case 472) with a *FIP1L1::RARA* fusion were submitted to the workshop. As they phenotypically resemble JMML but lack a *RAS* pathway mutation, they were classified by the panel as MDS/MPN with JMML‑like features.

**Fig. 6:**
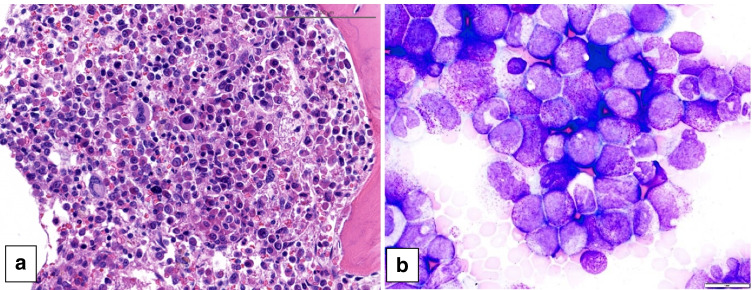
Myelodysplastic/myeloproliferative neoplasm, with JMML-like features and t(4;17)(q12;q21); *FIP1L1::RARA* fusion in a 9-month-old girl (case 471). The bone marrow biopsy (**a**) shows a markedly increased and left-shifted myelopoiesis, decreased erythropoiesis and dysmegakaryopoiesis. The bone marrow aspirate smear (**b**) demonstrates the expanded population of myelomonocytic cells and atypical promyelocytes with occasional basophilic granules, without Auer rods

Case 471 describes a 9-month-old boy presenting with fevers and recurrent otitis media. It has recently been published [57]. The PB showed anemia (Hb 8.8 g/dL), leukocytosis (45.9 × 10^9^/L), monocytosis (14%), increased promyelocytes (16%) without Auer rods, and numerous maturing myelomonocytic cells. There was marked dysgranulopoiesis and the monocytes showed some abnormal features. HbF was mildly elevated. Marked splenomegaly was present. No bruising, other bleeding manifestations, or features of disseminated intravascular coagulation were noticed. The BM biopsy and aspirate showed normocellular marrow with increased myeloid to erythroid ratio, markedly increased and left-shifted myelopoiesis, decreased erythropoiesis, and dysmegakaryopoiesis (Fig. 6a). There was an expanded population of myelomonocytic cells (29%) and promyelocytes (44%) with occasional basophilic granules (Fig. 6b). No Auer rods were identified. Ancillary studies demonstrated an in-frame fusion between *FIP1L1* exon 13 and *RARA* exon 3 resulting in a t(4;17)(q12;q21). In addition, a somatic alteration of *MAP2K2* p.R231L (VAF: 24.4%) was detected, on which no functional data are available and which may alter RAS-RAF-MEK-ERK signaling. Germline testing detected an *NF1* mutation, which could be a benign polymorphism. He achieved remission after treatment with ATRA and arsenic trioxide (ATO) combined with idarubicin which induced only a short-lived remission. SCT showed good outcome.

The second case (case 472) describes a 16-month-old girl with a 1-month history of intermittent fevers, generalized malaise, and night sweats. Her PB showed marked leukocytosis (156.4 × 10^9^/L) with neutrophilic left-shift, dysgranulopoiesis, atypical monocytes, and promyelocytes without Auer rods. She had no splenomegaly. The BM showed cellular marrow (> 90% cellularity) with marked and abnormal myelomonoytic hyperplasia and decreased erythropoiesis and megakaryopoiesis. Blasts were < 5%. Her karyotype was 46,XX,t(2;3)(q11.2;p21),t(4;17)(q12;q21),add(7)(p21),del(90(p21)[18]/46,XX[2]. A *FIP1L1::RARA* fusion was demonstrated as well as a bi-allelic deletion of *CDKN2A/2B*.

Myeloid neoplasms with a *FIP1L1::RARA* fusion have previously been described in two adults and a child [58–60]. They characteristically show an increase in promyelocytes. The two adult patients with *FIP1L1::RARA* fusion were diagnosed as acute promyelocytic leukemia (APL) [58, 60]. One of the cases was shown to be sensitive to all-trans retinoic acid (ATRA) [60]. Promyelocytes with Auer rods and disseminated intravascular coagulation, which are characteristic features of APL, were, however, lacking. The only previously described pediatric case with *FIP1L1::RARA* fusion showed a clinical picture most consistent with JMML [59].

Besides cases associated with *FIP1L1::RARA*, other JMML mimics include cases with rare rearrangements, like *ALK* and *ROS1* or *CCDC88C::FLT3* fusions [61]. Patients with suspected JMML who lack canonical RAS pathway mutations should have RNA-Seq performed to identify potentially targetable gene fusions (e.g., *FLT3*).

## Myelodysplastic syndromes

Pediatric MDS is rare, with an incidence of 1.8–4 cases/million [62]. It can occur at any age, with a median age at diagnosis of 8.3 years [63]. In contrast to adults, in whom low-grade MDS usually presents only with anemia, low-grade MDS in childhood commonly presents also with thrombocytopenia and neutropenia [63]. In addition, the mutational landscape of MDS in children differs from that in adults; mutations in genes for epigenetic regulation (e.g., *TET2*, *ASXL1*, and *DNMT3A*) and RNA splicing (e.g., *SF3B1* and *U2AF1*) are rare in pediatric primary MDS (2%), whereas mutations in genes from the RAS/MAPK pathway are common (45%) [64].

A relatively high percentage of pediatric MDS cases represent secondary MDS. This includes therapy-related myeloid neoplasms, MDS secondary to acquired aplastic anemia, and MDS secondary to inherited predisposition syndromes, including the inherited bone marrow failure syndromes Fanconi anemia, severe congenital neutropenia, dyskeratosis congenita, and Shwachman-Diamond syndrome.

Underlying inherited or de novo germline predisposing genetic aberrations are being recognized in an increasing amount of pediatric MDS cases. According to the revised 2016 WHO classification, myeloid neoplasms with germline predisposition can be classified in three groups: (1) Myeloid neoplasms with germline predisposition without a pre-existing disorder or organ dysfunction, (2) myeloid neoplasms with germline predisposition and pre-existing platelet disorders, and (3) myeloid neoplasm with germline predisposition and other organ dysfunction, which includes myeloid neoplasm with germline *GATA2* mutation, myeloid neoplasms associated with bone marrow failure syndromes, myeloid neoplasms associated with telomere biology disorders, JMML associated with neurofibromatosis, Noonan syndrome or Noonan syndrome-like disorders, and myeloid neoplasms associated with Down syndrome [65]. Additional germline predisposing mutations are still being discovered, such as mutations in sterile alpha motif domain-containing protein 9 (*SAMD9*) and its paralogue, SAMD9-like (*SAMD9L*) [66, 67]. In patients with a hematologic malignancy, cultured skin fibroblasts are the preferred tissue for germline mutation testing.

Together, *GATA2* and *SAMD9/SAMD9L* syndromes currently form the most common predisposing conditions in pediatric MDS. In cases that originally were classified as primary pediatric MDS, 7% showed a *GATA2* mutation and 8–17% a *SAMD9/SAMD9L* mutation [64, 68, 69]. They are particularly found in the setting of monosomy 7 [64, 68].

GATA2-related MDS typically shows BM hypocellularity with multilineage dysplasia [70–72]. Dysplastic megakaryocytes in GATA2 deficiency characteristically are both large and small with separated and peripheralized nuclear lobes, which may provide a morphologic clue to an underlying GATA2 deficiency [70–72]. In addition, increased reticulin fibrosis is much more frequent in GATA2 deficiency than in de novo MDS (46–73% versus 10%) [70–72]. Cytogenetic abnormalities are common in patients with GATA2 deficiency, most common being monosomy 7, der(1;7), or trisomy 8, whereas complex karyotypes are generally not seen [68, 73].

SAMD9/9L syndromes are autosomal dominant syndromes with variable penetrance. They cause a spectrum of multisystem disorders that carry a markedly increased risk of developing myeloid malignancies with monosomy 7 [74]. The median age at diagnosis of MDS in SAMD9/9L syndromes is 10 years (0.2–17.6), with the vast majority (90%) showing a hypocellular marrow with features similar to RCC [69]. MDS with excess blasts is seen in 10% of cases [69].

RCC is a provisional entity dealt with in the WHO MDS chapter [75]. It is defined by persistent cytopenia with < 5% blasts in the BM and < 2% blasts in the PB. Most patients with RCC (61–67%) have normal cytogenetics [76]. The vast majority (81%) shows decreased cellularity with a marrow which superficially resembles aplastic anemia. In contrast to aplastic anemia, RCC is reported to show left-shifted erythropoiesis arranged in large erythroid islands with an increased number of immature erythroblasts, particularly proerythroblasts [77]. Micromegakaryocytes are present in some cases [76, 77]. A similar RCC-like morphological picture can be seen in some hematological and non-hematological diseases, which must be excluded before a diagnosis of RCC can be made. Hematological disorders include the inherited BM failure syndromes, paroxysmal nocturnal haemoglobinuria (PNH), and hepatitis-associated aplastic anemia. Non-hematological disorders include infections, vitamin deficiencies, and metabolic disorders. A minority (19%) of RCC cases shows normo- or hypercellular marrow. Similar to hypocellular RCC, the BM in normo- and hypercellular RCC typically shows enlarged erythroid islands with impaired maturation, which is essential and an important clue to the diagnosis of RCC [76, 77].

### Workshop cases

In total, 16 of the submitted cases were classified by the panel as MDS (Table 4).

**Table 4:** Myelodysplastic syndrome (MDS) cases submitted to the 2020 EAHP workshop

**Case #**	**Submitter**	**Age** years	**Sex**	**Hb** g/dL	**WBC** × 10^9^/L	**Plt** × 10^9^/L	**Panel diagnosis**	**Molecular aberrations/remarks**
689	Dr. Shestakova, Orange, USA	3	F	2.7	5.1	41	**MDS-U**	Complex cytogenetics, *LZTR1* mutation Rapid progression to AML
179	Dr. Coviello, Fort Sam Houston, USA	7	M	14.8	3.9	139	**MDS-U**	Normal karyotype. FISH: del7/7q (40%), WES: RRAS (in frame insertion). Sporadic vs. RASopathy/Noonan syndrome (?)
779	Dr. Nagrale, Abu Dhabi, United Arab Emirates	6	F	9.9	0.22	70	**MDS-U**	Del7
228	Dr. Al-Khatib, Irbid, Jordan	10	M	10	1.1	90	**MDS-U**	Molecular data not available History of congenital neutropenia (favor sec. MDS)
322	Dr. Kläger Vienna, Austria	15	F	6.0	5.3	132	**MDS-SLD-F**	Trisomy 8
682	Dr. Laximanarayana, Udupi, India	2	F	6.1	6.1	16	**MDS EB1-F**	11q translocation Rapid progression to AML
329	Dr. Sadigh, Boston, USA	8	F	n/a	n/a	60	**MDS-EB2-F**	Karyotype normal, 3rd biopsy FISH only: trisomy 15
754	Dr. Leventaki, Winsconsin, USA	18	F	7.1	3.9	256	**MDS-U-F**	Trisomy 8
723	Dr. Wooldridge, New York, USA	18	M	9.3	1.7	23	**MDS-U-F**	Monosomy 7 Prior long history of neutropenia and thrombocytopenia, favor sec. MDS. Donor cell MDS post CB-SCT
545	Dr. Petrushevska, Skopje, Macedonia	8	F	n/a	n/a	n/a	**MDS-EB2**	Pancytopenia Del7q
503	Dr. Slonim, Chicago, USA	7	M	7.6	7.7	57	**MDS-EB2**	*NRAS* missense mutation in subsequent AML
525	Dr. Kaur, Chicago, USA	13	F	8.6	3.0	60	**MDS-EB2**	*WT1* L378fs*70 mutation
677	Dr. Tashkandi, Pittsburgh, USA	7	M	11.9	2.2	38	**MDS-EB2**	t(3;5)(q25;q35); *MLF1::NPM1*, which is seen in younger pediatric patients with AML/MDS–excellent response to SCT
726	Dr. Tashkandi, Pittsburgh, USA	8	M	8.3	1.8	26	**MDS-EB2 (1) evolving in AML-MRC (2)**	aUPD of 1p36.33-p35.5 additional del9p upon relapse *NUP98* rearr. in evolved AML
414	Dr. Rozenova, Rochester, USA	16	M	n/a	n/a	n/a	**t-MDS**	*KMTA::CREBBP* fusion *PTPN11* mutation
647	Dr. Saft, Stockholm, Sweden	14	F	n/a	n/a	n/a	**t-MDS**	Complex karyotype

*AML* acute myeloid leukemia; *AML-MRC* AML with myelodysplasia-related changes; *aUPD* acquired uniparental disomy; *CB* cord blood; *CB-SCT* cord blood stem cell transplantation; *Hb* hemoglobin; *MDS* myelodysplastic syndrome; *MDS-EB1/2* MDS with excess blasts 1 or 2; *MDS-SLD-F* MDS with single lineage dysplasia and fibrosis; *MDS-U* MDS, unclassifiable; *MDS-U-F* MDS-U with fibrosis; *t-MDS* therapy-related MDS; *n/a* not available; *rear* rearrangement; *plt* platelet count; *SCT* stem cell transplantation; *WBC* white blood count; *WES* whole exome sequencing

Seven MDS cases had excess of blast (1 × MDS-EB1, 6 × MDS-EB2) and two of them were accompanied by fibrosis. One of the MDS-EB2 cases (case 677) was a 7-year-old boy with a t(3;5)(q25;q35); *MLF1::NPM1*, which is an aberration typically seen in younger pediatric patients with MDS or AML and is associated with an excellent response to SCT.

There were two cases of therapy-related MDS; one case of a 16-year-old boy with a history of Ewing sarcoma at the age of 15, and one case of a 14-year-old girl with a history of spinal neuroblastoma at the age of 2.

**Fig. 7:**
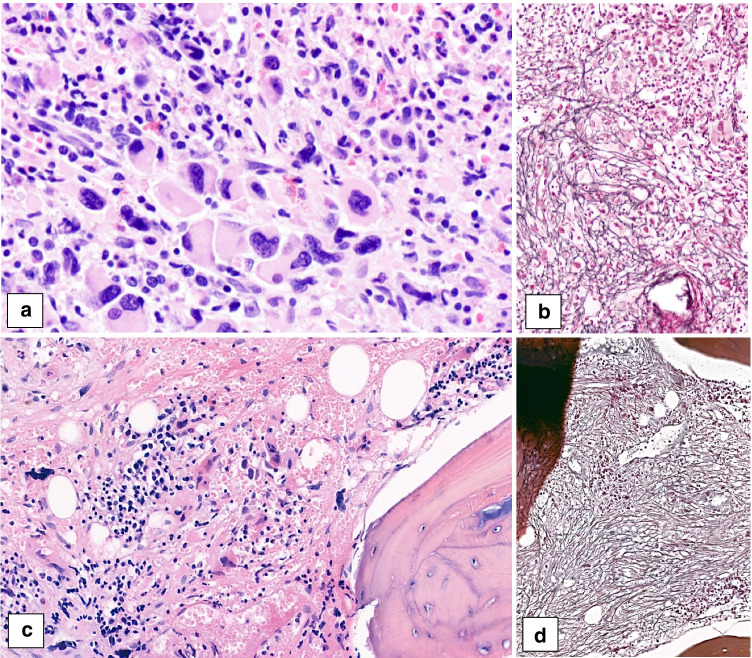
Two pediatric cases of myelodysplastic syndrome (MDS) with fibrosis showing pronounced dysplasia, espcecially in the megakaryocytes. **a**, **b** Case 754 (MDS, unclassifiable, with fibrosis) shows moderate fibrosis, MF2 (**a** HE, **b** reticulin stain). **c**, **d** Case 322 (MDS with single lineaged dysplasia with fibrosis) shows severe fibrosis, MF3 (**c** HE, **d** reticulin stain)

One of the submitted cases was classified as MDS with single lineage dysplasia and fibrosis (case 322) and six cases were classified as MDS, unclassifiable (MDS-U), based on the presence of single lineage dysplasia combined with pancytopenia (4 cases) or either single or multilineage dysplasia combined with 1% blasts in the PB (2 cases). Two of the MDS-U cases (case 723 and case 754) also showed fibrosis (MF2) and were therefore classified as MDS-U-F. Case 754 is illustrated in Fig. 7. In adults, MDS-U with 1% blasts has been described as a distinct subgroup of MDS-U with a poor prognosis [78]. Whether this also holds true for pediatric MDS-U remains to be determined.

In two of the MDS cases above (case 228 and case 723), the patients had a history of cytopenia, suggestive of an underlying germline condition: case 228 was a 10-year-old boy with MDS-U, known with congenital neutropenia, and case 723 was an 18-year-old young man with MDS-U-F with monosomy 7, who had a long history of neutropenia and thrombocytopenia. The latter patient received an allogeneic matched unrelated donor cord blood transplantation, 3 months after which he developed a donor cell MDS with again monosomy 7.

In one of the MDS-U cases (case 179), the 7-year-old boy had a de novo heterozygous mutation of *RRAS* and a concurrent del7/7q detected in his myeloid neoplasm. The presence of multiple phenotypic abnormalities, including scoliosis, craniosynostosis, and cardiac anomalies, as well as delayed milestones, was suggestive of an underlying RASopathy (i.e., Noonan syndrome), but the fact that his *RAS* mutation was not detected by whole exome sequencing (trio exome analysis) that was performed on normal tissue to detect germline mutations was in favor of a somatic mutation.

None of the cases was felt by the panel to fit the diagnosis of RCC; these cases were at least moderately cellular, not displaying the typical low cellularity of RCC reminiscent of AA. They also did not display markedly enlarged erythroid islands with impaired maturation, which is a key feature for the diagnosis of RCC, including the normo- and hypercellular RCC variants [76]. Rather, a predominance of granulopoiesis and a picture more consistent with MDS in adults was seen. One of the submitted cases came close to hypocellular RCC, but was eventually felt by the panel to represent aplastic anemia, partly due to its clinical picture with rapid onset cytopenia and the absence of cytogenetic aberrations.

Interestingly, as mentioned before, 5 of the 16 MDS cases showed marrow fibrosis (Fig. 7). The mean age in these fibrotic cases was 12.2 years (range 2–18 years), with a male:female ratio of 1:4. All of the workshop MDS-F cases showed chromosomal aberrations: 2 cases showed trisomy 8 (case 322 classified as MDS-SLD-F and case 754 classified as MDS-U-F), 1 case showed 3 copies of *PML* suggestive of trisomy 15 (case 329 classified as MDS-EB2-F), 1 case had monosomy 7 (case 723 classified as MDS-U-F), and the fifth case (case 682 classified as MDS-EB1-F) showed on interphase FISH a translocation of chromosome 11q with unknown fusion partner. In literature, fibrosis in pediatric MDS has been associated with germline *GATA*2 mutations [70–72], but unfortunately the information on GATA2 status was not available in many of submitted cases.

## Acute myeloid leukemia

Although all types of AML can occur at the pediatric age, some forms occur more often or even exclusively in childhood, and the genetic landscape of pediatric AML differs from that of adult AML [79]. Certain mutations that are common in adult AML, such as mutations in *DNMT3A*, *TP53*, and *IDH1* or *IDH2*, are rare in pediatric AML [79]. Mutations in *NRAS*, *KRAS*, *KIT*, *WT1*, *MYC*, *CBL*, *GATA2*, *SETD2*, *PTPN11*, and *FLT3* are more common in children, and sites of mutation often differ from those detected in adults [79]. In addition, chromosomal rearrangements are more prevalent in childhood, with highest frequencies being found in infancy, declining with age [79].

The most common pediatric AML belongs to the group of AML with recurrent genetic abnormalities (AML-RGA). They include t(9;21)(p21.3;p23.3); *KMT2A::MLLT3* (20–22%), t(8;21)(q22;q22.1); *RUNX1::RUNX1T1* (9–15%), and inv(16)(p13.1q22) or t(16;16)(p13.1;q22); *CBFB::MYH11* (6–10%), of which t(8;21)(q22;q22.1); *RUNX1::RUNX1T1* and the two with involvement of chromosome 16 are associated with a favorable prognosis [79–81]. In cases of AML with variant *KMT2A* rearrangement, the prognosis depends on the fusion gene partner; some show a favorable prognosis, e.g., t(1;11)(q21;q23), while others show an adverse prognosis, e.g., t(10;11)(p12;q23), t(10;11)(p11.2;q23), t(6;11)(q27;q23), and t(4;11)(q21;q23) [82]. In t(9;11)(p22;q23); *KMT2A::MLLT3* (previously called *MLL-AF9* and the most common *KMT2A* fusion in pediatric AML), prognosis appears to be related to morphology, as those with acute monoblastic features have a significantly better outcome than cases showing other cytochemical types, at least in one study [82].

In addition to *KMT2A*, *RUNX1*, and *CBFB*, there are many other rare chromosomal rearrangements that display a higher prevalence in pediatric than in adult AML. Some rearrangements preferentially occur at infancy, such as AML (megakaryoblastic) with t(1;22)(p13.3;q13.1); *RBM15::MKL1*, AML with t(7;12)(q36;p13); *MNX1::ETV6*, AML with t(8;16)(p11;p13); *KAT6A::CREBBP* (which can spontaneously remit in infancy, but has an intermediate prognosis in later childhood), and AML with inv(16)(p13.3q24.3); *CBFA2R3::GLIS2* as reviewed by Quessada et al. [83]. Others typically present later in childhood (median age varying between 6.6 and 12.4 years), such as AML with t(10;11)(p12;q14); *PICALM::MLLT10*, AML with t(6;9)(p23;34.1); *DEK::NUP214*, AML with t(16;21)(p11;q22); *FUS::ERG*, AML with t(16;21)(q24;q22); *RUNX1::CBFA2T3*, AML with t(5;11); *NUP98::NSD1*, AML with t(6;11)(q27;q23.3); *KMT2A::MLLT4*, and AML with t(15;17)(q24.1;q21.2); *PML::RARA* [83]. Other rare cytogenetic AML subgroups have also been reported [83].

As already discussed above in the section on MDS, several inherited or de novo germline aberrations are known to predispose to the development myeloid neoplasms. One of the predisposition syndromes is Down syndrome (DS), which children have a markedly increased risk of developing AML in their first 5 years of life, especially acute megakaryoblastic leukemia (AMKL). MDS and AML in DS are biologically similar and are therefore grouped together under the diagnosis “myeloid leukemia associated with Down syndrome” [84]. Somatic mutations in *GATA1* are an early event in the leukemogenesis and are characteristic for myeloid leukemia associated with DS [85, 86]. Approximately 10% of newborns with DS develop a preleukemic clone with somatic *GATA1* mutation. This preleukemic clone, known as transient myeloproliferative disease (TMD) or transient abnormal myelopoiesis (TAM), may be morphological indistinguishable from Down-associated AMKL, but resolves spontaneously in 60% of cases at a mean of 58 days (range 2–194 days) [87]. About 20% subsequently develops leukemia, predominantly AMKL, and about 20% experience early death mainly due to liver failure [87].

### Workshop cases

In total, 12 cases of pediatric AML were included in the workshop, covering a wide spectrum of AML subtypes (Table 5). Four cases were classified as AML-RGA, four as AML-MRC, and three as AML not otherwise specified (AML-NOS). In addition, there were two cases of myeloid sarcoma.

**Table 5:** Acute myeloid leukemia (AML) and myeloid sarcoma cases submitted to the 2020 EAHP workshop

	**Submitter**	**Age**	**Sex**	**Genetics**	**Panel diagnosis**	**Remarks**
548	Dr. Leguit, Utrecht, the Netherlands	7 months	F	*53,XX, + der(1)t(1;22),t(1;22)(p13.3;q13.1), + 6, + 7, + 15, + 19, + 19, + 21[5]/53,XX, + der(1)t(1;22),t(1;22)(p13.3;q13.1), + 6, + 7, + 15, + 19, + add(19)(p13), + 21[2]/53,XX, + der(1)t(1;22),t(1;22)(p13.3;q13.1), + 6, + 7, + 15, + 19, + 21, + mar[5]	**AML (megakaryoblastic) with t(1;22)(p13.3;q13.1); *RBM15::MKL1***	
511	Dr. Slonim, Chicago, USA	3 years	F	*46,XX[7]/46,XX,der(10)t(1;10)(q21;q26)[13] *RNA analysis: CBFA2T3::GLIS2 fusion transcript *JAK2 mutation c.2049A > T (p.Arg683Ser), a variant of potential clinical significance (VAF n/a)	**AML with *CBFA2T3-GLIS2***	Equivalent to inv(16)(p13.3;q24.3) RAM phenotype (CD56 + , dim CD45 + , CD38-, HLA-DR-)
583	Dr. Loghavi, Houston, USA	12 years	M	*47,XY, + 10[1]/46,XY[29] *Mutations in *NPM1* (45%), *RAD21* (43%), *KRAS* (27%), *2 × *PTPN11* (10% and 2%)	**AML with mutated *NPM1***	
187	Dr. Ruskova, Auckland, NZL	10 years	M	*45,X,-Y, t(8;21)(q22;q22) *FISH: t(8;21); *RUNX1T1::RUNX1* *2 × *KIT* mutation: c.1251_1266delins16 and c.2466 T > A (p.Asn822Lys) (VAF n/a)	**AML with t(8;21); *RUNX1T1::RUNX1* (with myelomastocytic differentiation)**	AML with myelomastocytic differentiation. Two *KIT* mutations, not described in SM
282	Dr. Sadigh, Boston, USA	10 months	F	*46,XX[20] *FISH: *KMT2A (MLL*) at 11q23 (partner not identified)	**AML-NOS (megakaryoblastic)**	Karyotype: normal. FISH: *KMT2A* (*MLL*) at 11q23 (partner not identified)
766	Dr. Hussein, New York, USA	9 years	F	*46,XX,der(4)t(4;8)(q32;q13)[5]/49,idem,del(6)(q13q21), + 9, + 15, + 20[1]/46,XX[14] *FISH: 3 copies *RUNX1T1* and *MYC* with 2 copies of centromere 8 *FLT3-ITD, and *WT1, NRAS *(subclonal),* PTPN11 *(subclonal) mutations (VAF n/a)	**AML-NOS**	Presence of *FLT3-ITD* and *WT1* mutation
247	Dr. Wang, San Francisco, USA	11 weeks	F	*47,XX,t(1;8)(p32;q22), + 19[11]/46,XX[11] *FISH: gain *RUNX1T1 (*40,5%) *NGS: *KIT* p.D816Y (9%)	**AML-NOS**	Rich in proerythroblasts but not fulfilling the criteria for pure erythroid leukemia
361	Dr. Sadigh, Boston, USA	7 years	F	46,XX,t(3;3)(p24;q26.2),-16, + r[13]/46,sl,del(11)(q21q23)[7] *RNA fusion gene analysis: *TBL1XR1::RARB* fusion transcript	**AML-MRC (relapsed)**	Complex karyotype with t(3;3)(p24;q26.2); *TBL1XR1::RARB*. Resembles APL but is ATRA insensitive. Original AML type unknown
796	Dr. Kumar, Memphis, USA	5 years	F	*46,XX,del(7)(q22q36) [16/20]; 46,XX [4/20] *NGS: 46,XX,t(3;7)(q26.2;q21.2); *CDK6::MECOM;* -17q21.2q36.3; + 3q36.2q29 t(3;7)(q26.2;q21.2); FLT3 A680V mutation *cnLOH: 17q21.31q25.3	**AML-MRC**	Del(7)(q22); WGS: t(3;7)(q26.6;q21.2) WGS, *CDK6::MECOM*
773	Dr. Reszec, Bialystok, Poland	12 years	F	*46,XX[20]	**AML-MRC**	AML-MRC ex MDS (MDS-MLD)
150	Dr. Marcus, Boston, USA	2 years	F	*47,X,del(X)(q22),add(4)(q31,3), + 6,add(6)(q21),add(6)(q23),der(7)t(7;7)(p15;q11.2),inv(16)(p11.2q13),inv(17)(p11.2q23),add(20)(q13.3)[7]/ 46,XX[13] *FISH: 3 copies of *CDKN2A* *JAK2 V617F (6.2%)	**AML-MRC**	Complex karyotype with megakaryoblastic differentiation
522	Dr. Liu, Foshan, China	3 months	F	No molecular studies performed	**Myeloid sarcoma**	Located in the skin Monoblastic variant
630	Dr. Katerji, Rochester, USA	5 years	M	*46,XY,t(8;20;21)(q22;q11.2;q22)[19]/46,XY[1] *FISH: *RUNX1T1::RUNX*	**Myeloid sarcoma**	Orbital mass. Followed by AML t(8;20;21)(q22;q11.2;q22); *RUNX1::RUNXT1*
523	Dr. Gasljevic, Ljubljana, Slovenia	12 months	F	*47,XX,t(11;12)(q22;p13), + 22[1]/47,idem,del(4)(q21q27)[2]/47,sdl1,del(6)(q21q26)[2]	**MPAL (T/Myeloid) versus AML-NOS (acute megakaryoblastic leukemia)**	

*AML-MRC* AML with myelodysplasia-related changes; *AML-NOS* AML, not otherwise specified; *SM* systemic mastocytosis; *APL* acute promyelocytic leukemia; *MDS* myelodysplastic syndrome; *MDS-MLD* MDS with multilineage dysplasia; *MPAL* mixed phenotype acute leukemia; *n/a* not available; *WGS* whole genome sequencing

#### AML with recurrent genetic abnormalities

The first AML-RGA case (case 458) was a 7-month-old girl with AML (megakaryoblastic) with t(1;22)(p13.3;q13.1); *RBM15::MKL1*. This is a rare translocation occurring in infants and presenting as AMKL.

The second case (case 511) was a 3-year-old girl with AML with *CBFA2T3::GLIS2*, which is equivalent to inv(16)(p13.3;q24.3). This is a rare cryptic chromosomal translocation associated with treatment-refractory disease, occurring especially in infants, and presenting in 20% of cases as an AMKL. In this case, the blasts showed a so-called RAM phenotype (phenotype named after initials of the first patient in which this was described), consisting of bright CD56 expression, dim or negative CD45 and CD38 expression, and lack of HLA-DR expression. The RAM phenotype is mainly seen in infants and very young patients, and an independent risk factor in pediatric AML patients associated with extremely poor outcome [88].

The third case (case 583) was a 12-year-old boy with AML with mutated *NPM1*. The *NPM1* mutation is one of the most common recurrent cytogenetic aberrations in AML, and, in contrast to the previous two cytogenetic aberrations, not specific for childhood. Its frequency is rare in AML in infants (2.5%), but it increases with age to up to 34% in adult AML [79]. In addition to the *NPM1* mutation, the patient had several other mutations: *RAD21* (VAF: 43%), *KRAS* (VAF: 27%), and two different *PTPN11* mutations (VAF: 10% and 2%).

The fourth AML-RGA case (case 187) was a 10-year-old boy with AML with t(8;21)(q22;q22.1); *RUNX1T1::RUNX1*, which interestingly showed myelomastocytic differentiation with increased serum tryptase (29 μg/L) and two *KIT* mutations (c.1251_1266delins16 and c.2466 T > A (p.Asn822Lys)), which have not been described in systemic mastocytosis. In both the AML blasts and the mast cells, the t(8;21) could be detected, indicating they belong to the same clone.

#### AML with myelodysplasia related changes

Four of the submitted AML cases were classified by the panel as AML-MRC; one based on a previous history of MDS (case 773), two based on the presence of a complex karyotype (case 361 and 150), and the fourth (case 796) based on the presence of an MDS-related cytogenetic abnormality, being del(7)(q22). The latter case also showed a *CDK6::MECOM* aberration, which is a rare anomaly described in high-risk pediatric AML. The resulting overexpressed MECOM protein could be demonstrated by immunohistochemistry.

#### AML not otherwise specified

Three of the submitted AML cases were classified as AML-NOS. The first case (case 282) showed megakaryoblastic differentiation and a normal karyotype, but on FISH a *KMT2A* (*MLL*) translocation (fusion partner not identified).

The second AML-NOS case (case 766) had a *FLT3-*ITD and a *WT1* mutation, which concurrent presence forms a poor prognostic factor in de novo pediatric AML [89].

The third case (case 247) was considered AML-NOS. Although areas rich in proerythroblasts were noticed, the overall presence of > 20% myeloblasts supported a diagnosis of AML-NOS (over one of pure erythroid leukemia).

#### Myeloid sarcoma

There were two cases of myeloid sarcoma (Table 5), of which case 522 was a myeloid sarcoma (monoblastic variant) occurring in the skin of a 3-month-old girl, and case 630 a myeloid sarcoma in the orbit of a 5-year-old boy, which was followed by an AML with t(8;20;21)(q22;q11.2;q22.1); *RUNX1::RUNXT1*.

#### Acute megakaryoblastic leukemia

**Fig. 8:**
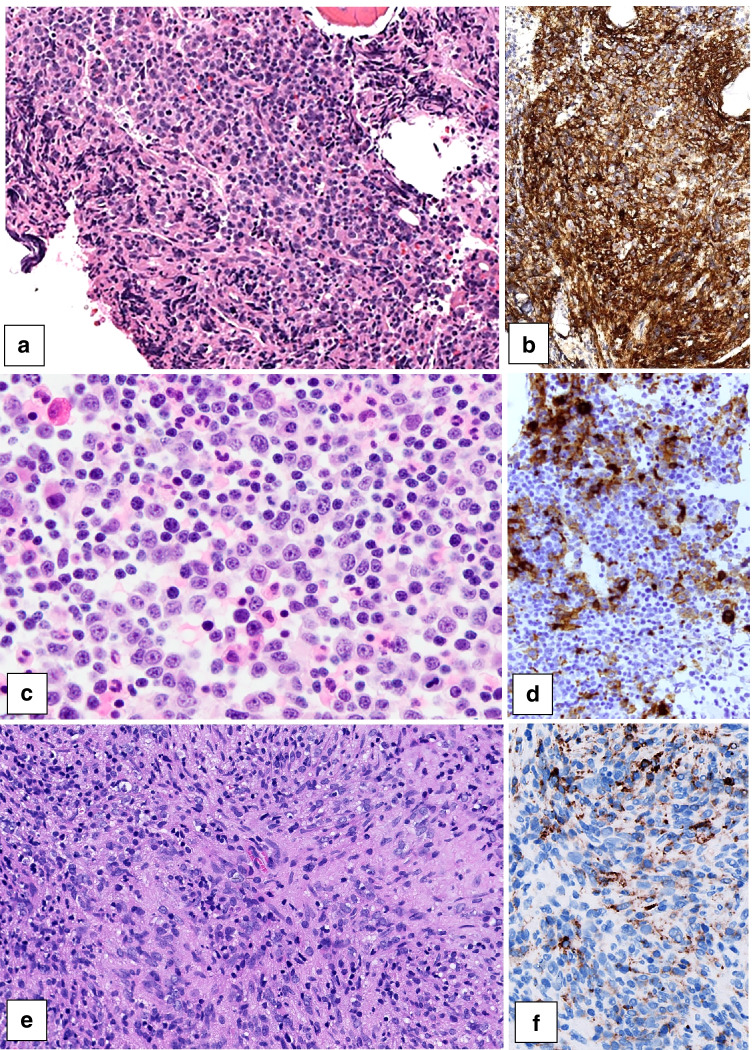
Pediatric bone marrow biopsies showing acute myeloid leukemia (AML) with megakaryoblastic differentiation: **a**, **b** Case 282, classified as AML-NOS (megakaryoblastic) with *KMT2A* rearrangement (**a** HE, **b** CD61); **c**, **d** case 150, classified as AML-MRC due to a complex karyotype, showing partial megakaryoblastic differentiation (**c** HE, **d** CD61); **e**, **f** case 458, classified as AML-RGA, being an example of an AML (megakaryoblastic) with t(1;22)(p.13.3-q13.1); *RBM15::MKL1* (**e** HE, **f** CD61)

Interestingly, among the submitted AML cases, there was a relatively high frequency of AMKLs (Fig. 8), none of which associated with DS. Non-Down syndrome AMKL can be classified according to the WHO classification as AML-RGA, AML-MRC, or AML-NOS. Case 458 is an example of AML-RGA, being an AML (megakaryoblastic) with t(1;22)(p.13.3-q13.1); *RBM15::MKL1*. Other commonly recurring rearrangements in non-Down syndrome pediatric AMKL are *CBFA2T3::GLIS2*, *NUP98::KDM5A*, *HOX* rearrangements, and *KMT2A* rearrangements, the latter being illustrated by case 282. These other recurring rearrangements are, however, not separate WHO entities but currently classified as AML-NOS (megakaryoblastic), although they do have prognostic value as outcomes vary considerably [90]. Case 150 shows an example of AMKL classified as AML-MRC due to its complex karyotype.

## Summary

The first section of the BM workshop of the *EAHP 2020 virtual meeting* was dedicated to pediatric myeloid neoplasms. Several interesting “take home messages” can be drawn from it:

- CML is rare in childhood and CML biology in adults and children is different as underlined by the different BCR breakpoint distribution seen in the latter. One must always be aware of cytogenetically cryptic *BCR::ABL1* variants and *ABL1* TKI resistance mutations, as illustrated by the workshop cases 505 and 744.
- Pediatric *BCR::ABL1*-negative MPNs are rare and differ from the adult cases at several points:
  * Mild splenomegaly is common even in ET and should therefore not be used as a criterion for separating ET from PMF.
  * Thrombotic events are less frequent than in adults and are largely venous, with a high frequency of Budd-Chiari syndrome.
  * High cellularity and lack of iron storages are common, especially in young children, and are therefore less reliable criteria for the distinction of ET versus PV.
  * WHO diagnostic criteria for diagnosing adult PV need to be adjusted in children by using appropriate age-related thresholds for establishing presence of erythrocytosis.
  * Although it has been reported that pediatric MPNs have a lower frequency of driver mutations (i.e., a higher frequency of triple negativity), this was not confirmed by the cases submitted to the workshop, in which a driver mutation was detected in 8 out of 11 (73%) cases. Further studies of adequately annotated cases diagnosed according to the WHO approach seem to be needed to clarify the exact frequency.
  * The features of pediatric PMF are overall similar to those seen in adult PMF, and the two submitted cases of prefibrotic (pre-)PMF underline the fact that that pre-PMF can also occur in children.
- Apart from JMML, the “adult types” of MDS/MPN are basically not seen at the pediatric age. The workshop cases illustrate the following:
  * JMML may present as AML-MRC.
  * *RRAS2* is one of the less frequently mutated genes of the RAS-pathway in JMML.
  * A second, often subclonal, mutation occurs in less than half of JMML cases; this mutation can occur in another RAS pathway gene or in *SETBP1*, *JAK3*, *SH3BP1*, *SH2B3*, or components of the polycomb repressive complex 2, including *EZH2* and *ASXL1.*
  * Some of the JMMLs occur in the setting of an underlying RASopathy, which is a group of developmental disorders characterized by typical phenotypic features, caused by a germline mutation in genes that encode components of the RAS/MAPK pathway. Of these, Noonan syndrome, neurofibromatosis type 1, and CBL syndrome predispose to JMML.
  * JMML is generally an aggressive disease but milder forms and transient myeloproliferations occur: (1) Few cases with a *KRAS* or *NRAS* mutation show an indolent clinical course and long-term survival, which entity is called RAS-associated autoimmune leukoproliferative disorder (RALD), (2) infants with Noonan syndrome develop a transient JMML-like myeloproliferation in 2.5–10% of cases, which is almost invariably associated with a *PTPN11* mutation, (3) JMML with a germline *CBL* mutation may show spontaneous regression.
  * Two rare cases of MDS/MPN with JMML-like features and t(4;17)(q12;q21); *FIP1L1::RARA* fusion are described. These cases manifest with monocytosis and an abnormal myelomonocytic proliferation similar to JMML but may be lacking some clinical features of JMML and do not contain identifiable mutations in the RAS pathway or other JMML defining genetic aberrations. *FIP1L1::RARA* fusion could also raise the possibility of acute promyelocytic leukemia, although typical features are lacking (no increase of promyelocytes with Auer rods, no disseminated intravascular coagulation). Whether these pediatric cases should be classified as JMML in the future remains to be determined.
- Pediatric MDS is rare. Most of the workshop cases were classified as MDS-U or MDS-EB1 or 2:
  * Five of the 16 MDS cases submitted to the workshop had fibrosis, with a female predominance (M:F = 1:4) and a mean age of 12.2 years (range 2–18 years). All fibrotic cases showed chromosomal aberrations, including trisomy 8, trisomy 15, monosomy 7, and 11q translocation.
  * Especially in children and young adults, MDS and AML may develop secondary to aplastic anemia, an inherited bone marrow failure syndrome or in the context of another underlying germline mutation which predisposes to the development of a myeloid neoplasm, of which *GATA2* and *SAMD9/SAMD9L* are at the moment the most common.
- All types of AML can occur at the pediatric age, and several cases of AML-RGA, AML-MRC, and AML-NOS, as well as 2 cases of myeloid sarcoma, were included in the workshop:
  * Some recurrent cytogenetic aberrations are typical of childhood, such as AML (megakaryoblastic) with t(1;22)(p13.3;q13.1); *RBM15::MKL1* and AML with *CBFA2T3::GLIS2* (equivalent to inv(16)(p13.3;q24.3)) as illustrated by the workshop. Others are more common in adults, but can be seen in childhood as well, as illustrated by a case of AML with t(8;21); *RUNX1T1::RUNX1*.
  * The workshop contained a relatively high frequency of AMKL, not associated with DS. These can be classified as AML-RGA, AML-MRC, or AML-NOS. The t(1;22)(p13.3;q13.1); *RBM15::MKL1* is the only recurrent cytogenetic abnormality in AMKL classified by the WHO as a separate entity, but the other recurrent cytogenetic aberrations in non-Down syndrome AMKL are also of prognostic importance and therefore deserve further investigation.
